# EHMTI-0079. Reduced volume of anterior thalamic nuclei in migraineurs

**DOI:** 10.1186/1129-2377-15-S1-F17

**Published:** 2014-09-18

**Authors:** S Magon, A May, A Stankewitz, PJ Goadsby, AR Tso, M Ashina, FM Amin, CL Seifert, M Chakravarty, J Müller, T Sprenger

**Affiliations:** 1 Department of Neurology, University Hospital Basel, Basel, Switzerland; 2 Institute for Systems Neuroscience, University of Hamburg, Hamburg, Germany; 3 Headache Group-Department of Neurology, University of California, San Francisco, USA; 4 Danish Headache Center and Department of Neurology Glostrup Hospital, University of Copenhagen, Copenhagen, Denmark; 5 Department of Neurology, Technische Universitaet Muenchen, Muenchen, Germany; 6 Campbell Family Institute CAMH, University of Toronto, Toronto, Canada

## Introduction

Previous studies on small samples of migraineurs have suggested subtle abnormalities of thalamic anatomy and function.

## Aims

To study changes of thalamic volume and shape in a large cohort of migraineurs using a novel label-fusion based segmentation approach.

## Methods

High-resolution 3D T-weighted MRI data were acquired at 3T in 131 migraineurs (31±9 years old; 109 women; monthly attack frequency: 3.2± 2.5; disease duration: 14±8.4 years) and 115 matched healthy subjects (29±7 years old; 81 women) at four international centers. The thalamus and thalamic subnuclei were segmented using a fully-automated multi-atlas approach. Deformation-based shape analysis was performed to localize thalamic surface abnormalities. To investigate group effects, ANCOVA was used (age, gender, site and brain volume as nuisance covariates; results were FDR corrected for multiple comparisons at threshold of 0.05.

## Results

We found volume reductions of the anterior, central, lateral dorsal (all FDR p<0.05) and a trend in the lateral posterior nucleus (FDR p<0.1) in migraineurs compared to controls. Patients with MwoA had by trend smaller volumes of anterior, central and lateral dorsal (all FDR<0.1) nuclei compared to controls. No significant differences were observed in patients with MwA compared with controls. No thalamic surface changes (shape analysis) and no relationship between thalamic volumes and migraine disorder duration or headache frequency was observed.

## Conclusions

The thalamic nuclei with abnormal volumes are densely connected to the limbic system. The data hence lend support to the view that higher-order integration systems are altered in migraine. No abnormalities were found in classical somatosensory nuclei.

No conflict of interest.

